# AlignWise: a tool for identifying protein-coding sequence and correcting frame-shifts

**DOI:** 10.1186/s12859-015-0813-8

**Published:** 2015-11-09

**Authors:** Teri Evans, Matthew Loose

**Affiliations:** School of Life Sciences, University of Nottingham, Nottingham, NG7 2UH UK

**Keywords:** Frame-shift, Protein-coding, Homology, Open reading frame, Genewise

## Abstract

**Background:**

Identifying protein-coding genes from species without a reference genome sequence can be complicated by the presence of sequencing errors, particularly insertions and deletions. A number of tools capable of correcting erroneous frame-shifts within assembled transcripts are available but often do not report back DNA sequences required for subsequent phylogenetic analysis. Amongst those that do, the Genewise algorithm is the most effective. However, it requires a homology wrapper to be used in this way, and here we demonstrate it perfectly corrects frame-shifts only 60 % of the time.

**Results:**

We therefore created AlignWise, a tool that combines Genewise with our own homology-based method, AlignFS, to identify protein-coding regions and correct erroneous frame-shifts, suitable for subsequent phylogenetic analysis. We compared AlignWise against other open reading frame finding software and demonstrate that the AlignFS algorithm is more accurate than Genewise at correcting frame-shifts within an order. We show that AlignWise provides the greatest accuracy at higher evolutionary distances, out-performing both AlignFS and Genewise individually.

**Conclusions:**

AlignWise produces a single ORF per transcript and identifies and corrects frame-shifts with high accuracy. It is therefore well suited for analysing novel transcriptome assemblies and EST sequences in the absence of a reference genome.

## Background

As sequencing technologies continue to improve, the number of transcriptome projects derived from species without a reference genome is increasing [1]. However, the absence of a reference genome makes identification and annotation of assembled transcripts challenging [2]. A typical RNAseq experiment will yield millions of reads, and after de-novo assembly the contig count is typically many-fold higher than the expected number of genes. One approach to reduce this number is to identify the subset of contigs containing an open reading frame (ORF). These protein-coding sequences can then be further analysed, for example by building phylogenetic trees, assessing rates of substitution and comparing levels of gene expression [1, 3]. Many of these bioinformatics experiments, particularly phylogenetic tree building, require an accurate protein-coding DNA sequence. This is vital for codon-based models of substitution, which require an in-frame sequence, and are more effective than either DNA or protein-based models [4, 5]. For species without a reference genome this is problematic as EST sequences and those assembled from next-generation techniques are known to contain sequencing errors leading to frame-shifts [6]. Indeed, a recent non-model vertebrate transcriptome project identified 3,618/14,471 (25 %) transcripts to contain a frame-shift [7], while a planarian transcriptome assembly identified an estimated frame-shift rate of between 4.2 %-13 % [8]. There is therefore a clear requirement for ORF finding software to accurately correct frame-shifts and produce a DNA sequence from transcripts, irrespective of their sequencing or assembly origins. This is particularly important when the provenance of available transcripts is unknown or the raw reads are unavailable.

There are many programs available that identify putative open reading frames within assembled transcripts but cannot correct frame-shifts, such as TransDecoder [9], OrfPredictor [10] and GetORF [11]. Many other methods, such as BLASTx [12] and ESTwise [13], can correct frame-shifts but do not produce a DNA sequence and as such are unsuitable for subsequent phylogenetic analysis. One method that does produce a DNA sequence is ESTscan [14], which is designed to identify ORFs and correct frame-shifts using a hidden Markov model (HMM). This requires a large quantity of known protein coding sequences, preferably from the same species [15], which is often unfeasible for species without a reference genome. Prot4EST attempted to solve this problem by building an HMM based on a modelled transcriptome [15], however one of the dependencies, DECODER, is now unavailable. It therefore relies on ESTscan, which we show below to be inaccurate, producing false positive results. Some alignment programs such as Genewise [13] and MACSE [16] can be used to correct frame-shifts but these require a homology wrapper to assess if the transcript is protein coding as well as to identify putative homologs. Although homology wrappers are provided with Genewise they require deprecated BioPerl modules. As we show below, with appropriate homology wrappers Genewise perfectly corrects a frame-shift only 60 % of the time. Furthermore, both Genewise and ESTscan produce multiple ORFs per transcript with no prioritisation as to which ORF is the most reliable. We also noted several potential improvements in homology based recognition that could be exploited and so developed a program that would produce a single ORF per transcript, accurately correct frame-shifts with minimal false positives and output a DNA sequence suitable for subsequent phylogenetic analysis.

AlignWise uses homology to identify contigs representing biologically relevant protein-coding sequences, and correct frame-shifts using two algorithms, AlignFS and Genewise. The AlignFS method uses a combination of BLAST searches [12] and multiple alignments using MUSCLE [17]. Here we describe the methodology behind AlignFS, and assess it’s ability to identify ORFs and correct frame-shifts in comparison to other software. We show that by combining AlignFS and Genewise we achieve fewer false positives than alternative methods or either approach alone. AlignWise also corrects frame-shifts with high accuracy, irrespective of evolutionary distance. AlignWise is designed to work on transcript sequences from any source, regardless of the method of generation.

## Implementation

**Fig. 1:**
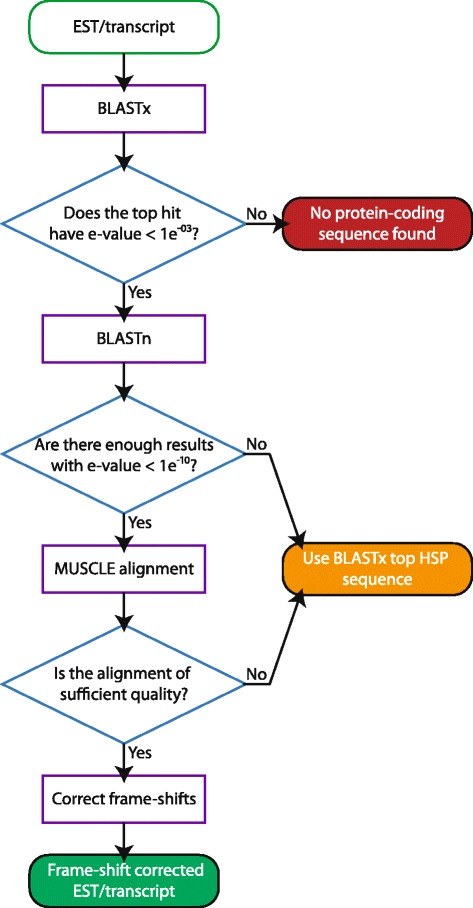
AlignFS workflow. The flowchart depicts the running order and logical progression within AlignFS

The AlignFS algorithm functions by identifying homologs, constructing a multiple alignment, and then correcting any identified frame-shifts (Fig. 1). An initial BLASTx search is used to identify putative protein-coding regions. If the top hit has an e-value less than or equal to 1E-03 then the transcript is considered protein coding, others are discarded. For top hits with multiple high-scoring segment pairs (HSPs), the putative ORF is considered to go from the earliest start point, to the furthest end point even if the HSPs are non-overlapping. To assess whether the ORF contains a frame-shift, the whole nucleotide sequence is run through BLASTn against a coding sequence database to identify homologs with an e-value less than 1E-10. By default, AlignFS searches for three homologs but this can be increased in the program settings. The identified homologs and whole nucleotide sequence are then globally aligned together using MUSCLE.

**Fig. 2:**
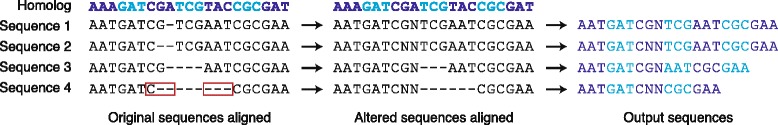
Demonstration of how AlignFS corrects frame-shifts caused by deletions. For each alternative situation the original sequences are shown aligned to a homolog, how they are altered and the final ORFs in frame. Sequence 1 has a single gap, which is corrected by adding an ‘N’. Sequence 2 shows a 2 bp gap, corrected by adding 2 ‘N’s. Sequence 3 has a 4 bp gap, which is treated as a single gap followed by an in-frame deletion, as such it is corrected as in Sequence 1. The final example shows a longer gap where each end is treated in accordance to the reference frame (red boxes), as such the first codon is treated as in Sequence 2, and the end section is deemed in-frame. Alternating codons are shown in different shades of blue

The multiple alignment is examined for gaps consistent with the presence of a frame-shift, i.e. not divisible by three. ‘N’s are added to replace transcript gaps (Fig. 2). For single or double spaced gaps the appropriate number of bases are added (Sequences 1 and 2, Fig. 2). Four base gaps are treated as single and in-frame 3 bp gaps (Sequence 3, Fig. 2). For longer gaps each end is processed individually according to the frame of one of the aligned coding sequences (Sequence 4, Fig. 2). In these cases a frame-shift correction could be made at either or both ends of the gap. For gaps conserved in all homologs (reflecting an insertion in the transcript) bases are removed from the transcript following the same logic as deletions. This accommodates situations where one of the aligned homologs contains a frame-shift as it will be ignored. Furthermore, any gaps that are less than 3 bp away from the start or end of the protein-coding region are skipped. If all the gaps not divisible by three are skipped then the transcript is considered as not having a frame-shift and the entire putative ORF returned.

If insufficient homologs can be identified or the alignment fails to pass thresholds on minimum alignment identity, maximum length of a gap or maximum percentage of gaps, no frame-shift corrections are attempted. Instead, the region from the top BLASTx HSP is selected as the putative ORF (Fig. 1). This is less likely to contain an unidentified frame-shift than an ORF based on multiple HSPs. As a consequence, for low quality alignments where a frame-shift may be ambiguous or poorly corrected, AlignFS will most likely provide a truncated in-frame sequence and not attempt a frame-shift correction. The running decisions made by AlignFS can be observed using the verbose option and the final decision per transcript is output to a log file.

After running the AlignFS algorithm, AlignWise will run Genewise using the same protein sequence identified in the BLASTx. Genewise is set to use the ESTwise algorithm and so does not search for introns within the transcript. The output from Genewise is parsed to stitch together each ORF, and then the longest ORF is selected. The protein sequence derived from this putative transcript is then compared against the AlignFS protein and the sequence with the best BLASTp result against the original BLASTx hit is selected. This decision process selects proteins aligned across their full length where possible, ensuring that AlignWise does not select sequences containing non-coding regions. The choice of output (AlignFS or Genewise) is written to the log file. Additionally, the choice of algorithm can be fixed within the running options to force use of either AlignFS or Genewise alone.

AlignWise can be run in parallel, which considerably speeds up the time to completion. Further speed improvements can be obtained by altering the BLASTx parameters to favor speed over sensitivity. AlignWise will optionally save all BLASTx results in an XML file, suitable for further analysis and annotation of sequences with packages such as Blast2Go [18]. It can also use previously identified orthologs, skipping the BLASTn step. Further options and information on the running parameters can be found in the release documentation. AlignWise is designed to be a flexible addition to most annotation and analysis pipelines.

### BLAST databases

AlignWise is provided with a small, vertebrate database, which is suitable for analyzing a range of species. This dataset contains protein-coding transcripts from the following Ensembl species (release 75, accessed June 2014): *Ciona savignyi, Danio rerio, Gallus gallus, Homo sapiens, Latimeria chalumnae, Lepisosteus oculatus, Mus musculus, Oryzias latipes, Pelodiscus sinensis, Taeniopygia guttata* and *Xenopus tropicalis*. This dataset was processed to remove the 198,624 coding sequences not beginning with an ATG codon, leaving 203,247 protein-coding sequences. A larger database is additionally supplied via FigShare (http://dx.doi.org/10.6084/m9.figshare.1245021), and comprises all vertebrate RefSeq mRNA sequences from the NCBI nucleotide database using TaxID 7742 (vertebrates), excluding those with ‘variant’ in the title (http://www.ncbi.nlm.nih.gov/nuccore/, downloaded August 2014). The 1,777,330 protein sequences were reduced to 936,009 using cd-hit [19]. To analyze how evolutionary distance affects AlignWise we subdivided this database (Table 1). Other BLAST databases can be used for AlignWise, and it is not required that the nucleotide and protein databases contain the same set of sequences. However, any nucleotide database must contain only coding-sequences in frame and both databases must be indexed using the ‘-parse_seq’ makeblastdb parameter.

**Table 1:** Summary of the subdivided BLAST databases

Database excludes	Minimum evolutionary distance (MYA)	No. of DNA sequences	No. of protein sequences
No species	0	1,777,330	936,009
Humans	6.3	1,765,559	935,166
Primates	92.3	1,556,336	855,115
Eutherians	162.6	655,629	415,776
Mammals	296.6	605,978	374,508
Amniotes	371.2	325,938	221,046
Tetrapods	414.9	295,722	199,882
Sarcopterygii	441	280,212	184,969

The minimum evolutionary distance is according to TimeTree [23]

## Results and discussion

### AlignWise is sensitive and able to reliably identify ORFs

We assessed the speed and sensitivity of AlignWise at identifying biologically relevant protein-coding sequences using four datasets, human ESTs, protein-coding rat cDNAs (NCBI), randomly generated DNA sequences from FaBox, constrained to a 50 % GC ratio [20], and randomly generated DNA sequences with no GC constraint. Each of these contained 1000 sequences. These acted as a model dataset with potential frame-shifts, a positive control and two negative controls respectively. These results were compared against other ORF finding software, namely ESTscan, OrfPredictor, and TransDecoder, as well as the AlignFS and Genewise algorithms on their own. Required dependencies for ESTscan are difficult to obtain for modern platforms, indeed, we were unable to install ESTscan on a recent Mac OSX platform. Therefore each program was compared on an Intel Core2-6320 within an Ubuntu 14.04 LTS environment compatible with all programs. TransDecoder was trained using 1000 human coding sequences and ESTscan was set to use a human HMM, otherwise all parameters were left as default. AlignFS, Genewise and therefore AlignWise were all set to use the default BLAST database provided, which contains human cDNAs but not ESTs or rat cDNAs. We attempted to compare the MACSE aligner, but this program proved too slow to use, taking more than 3 days to analyse 29/1000 Rat sequences.

AlignWise took the longest time to run using the standard settings, however, decreasing the BLASTx sensitivity and allowing AlignFS to make use of multiple cores considerably improves the runtime (Table 2). AlignWise, AlignFS and Genewise identified the same number of ORFs in the human and rat datasets and neither program identifies any ORFs within the randomly generated sequences. In contrast, although ESTscan ran fastest, it identified 835/1000 of the FaBox randomly generated, 50 % GC sequences as having putative open reading frames. Analyzing random DNA sequences with no GC constraint shows ESTscan to identify putative ORFs within the negative control, but at a reduced rate. Overall, ESTscan does not differentiate between randomly generated DNA and biologically relevant protein-coding regions but is affected by GC bias. OrfPredictor requires running BLASTx, which is reflected in a longer run time. However, it still attempts to generate putative ORFs for those sequences with no BLASTx result, and therefore identified 999 random sequences, irrespective of GC content, as being protein-coding. TransDecoder was trained using human coding sequences and so did not find any ORFs within the randomly generated sequences, however it found the fewest ORFs within the rat and human datasets, even when using a Pfam search. It is surprising that TransDecoder finds so few ORFs in the human ESTs given it is trained on human cDNA data. We presume this is a consequence of TransDecoder not considering frame-shifts in its algorithm.

**Table 2:** Comparing AlignWise and other ORF finding software

	Average	Number of ORFs found			
Program	run time	Human EST	Rat cDNA	FaBox DNA	Random DNA
AlignFS	02:12:54	633	990	0	0
Genewise	01:56:41	633	990	0	0
AlignWise	02:21:29	633	990	0	0
AlignWise (fast)	00:27:12	627	987	0	0
AlignWise (fastest)	00:03:46	627	987	0	0
ESTscan	00:00:01	526	971	835	366
OrfPredictor	01:26:06	995	1000	999	999
TransDecoder	00:00:17	299	924	0	0
TransDecoder (pfam)	00:37:11	339	950	0	0

AlignWise (fast) was set with the options ‘-a –T 2’, AlignWise (fastest) was run on a Mac 2 x 2.66 GHz 6-Core Intel Xeon using the option ‘-a –T 20’ to demonstrate the speed gains on a machine with more available CPUs. Only the longest ORF per starting transcript was counted in the TransDecoder and ESTscan outputs. The OrfPredictor run time includes running BLASTx

**Fig. 3:**
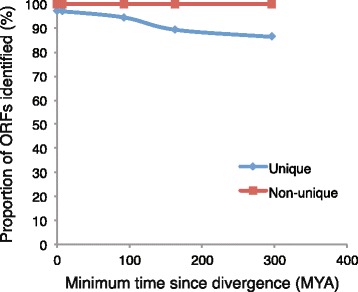
AlignWise can identify unique ORFs. The proportion of ORFs identified within unique and non-unique protein-coding sequences is shown for increasingly divergent databases

Although homology approaches are sensitive and have very few false positives, these methods will have high false negative rates if the ORFs are unique. We have assessed this by isolating the coding sequence of human genes with no known ortholog from Ensembl. These 2260 genes were processed with AlignWise using databases with increasingly divergent sequences, and compared against 2260 highly conserved genes with known orthologs in zebrafish. Using a non-human database identified 96 % of the unique ORFs, demonstrating that homology methods are capable of finding species-specific protein-coding genes (Fig. 3). The number of identified ORFs drops when using a BLAST database containing increasingly divergent species, but even at a minimum distance of 296 MYA, over 85 % of the unique ORFs were identified. This compares to the control dataset of 2260 genes with an ortholog in zebrafish, which showed that more than 99 % of the ORFs were identified, irrespective of the database.

### AlignFS is highly accurate at correcting frame-shifts

**Fig. 4:**
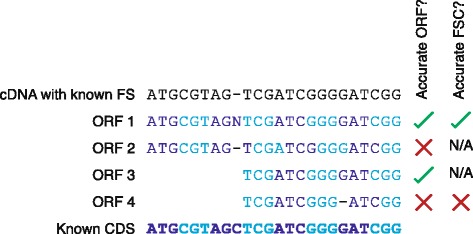
Four alternative ORF sequences. The cDNA with artificial frame-shift (FS) is shown along with four possible ORFs, all of which are aligned to the known CDS sequence. The first ORF has accurately corrected the frame-shift, producing an in-frame result. The second ORF has not made any changes to the input sequence and so is not in frame. The third ORF excludes the frame-shift position, but the resulting sequence is in-frame. The final ORF excludes the frame-shift position, but a frame-shift correction has still been made. Finally we show which of the ORFs are accurate, and then which of the sequences with an attempted FSC (ORFs 1 and 4) are accurate. Alternating codons are shown in different shades of blue

In-frame sequences are vital for phylogenetic analysis, and so we compared the accuracy of frame-shift corrections for AlignWise and ESTscan as well as AlignFS and Genewise individually. Each program analysed 1000 human protein-coding sequences artificially altered to include a random one-base frame-shift. None of the programs recaptured all 1000 ORFs from these sequences. AlignFS identified 974 ORFs, of which 765 were a perfect match to the coding sequence using the default database (Fig. 4, Table 3). Using this same protein database, Genewise produced 630 ORFs that were a perfect match to the CDS. AlignWise selected 457 of the AlignFS results and 289 of the Genewise ORFs; the remaining 228 sequences were identical between AlignFS and Genewise. AlignWise therefore identified 974 ORFs, of which 808 were aligned perfectly to the original cDNA, a higher value than AlignFS or Genewise achieved independently. Only 2 sequences were lost when using the faster and less sensitive BLASTx option. Using a large database based on the NCBI RefSeq collection, AlignFS identifies more ORFs (989) of which 942 are accurate while Genewise identifies 613 ORFs that are a perfect match to the CDS. AlignWise selected 471 of the AlignFS results and 229 of the Genewise results based on their BLASTp alignments. In total, 880 of the 989 ORFs were a perfect match to the CDS, a marginally worse result than AlignFS. ESTscan, using a human HMM, produced 259/963 accurate ORFs, with both ESTscan and Genewise tending to extend the ORF beyond the known coding region (Table 3).

**Table 3:** Comparing AlignFS, Genewise, AlignWise and ESTscan at producing ORFs

Program	Total	Accurate	Inaccurate	Mean CDS coverage (%)
AlignFS	974	765	209	87.43
Genewise	974	630	344	99.69
AlignWise	974	808	166	91.87
AlignWise (fast)	972	806	166	91.98
AlignFS (RefSeq)	989	942	47	96.57
Genewise (RefSeq)	989	613	376	104.36
AlignWise (RefSeq)	989	880	109	98.49
ESTscan	963	259	704	116.30

**Fig. 5:**
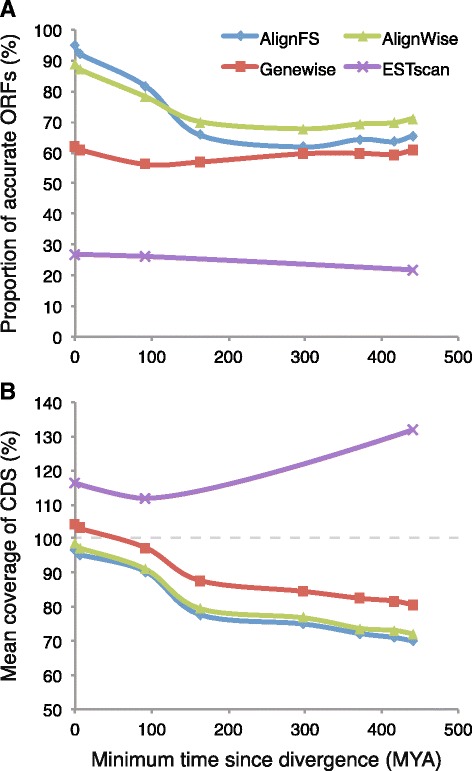
Measuring the affect of database divergence ORF quality. **a** The accuracy of ORF frame-shift correction is shown for the four methods using increasingly divergent databases. **b** The mean coverage of the CDS is shown using those same databases, a line is drawn at 100 % since no software should be finding ORF sequences longer than the known protein-coding region

Identifying ORFs from assembled transcripts of non-reference species would require using databases or HMMs containing dissimilar sequences. To simulate this we divided the RefSeq database into smaller databases containing increasingly divergent sequences, for example a database excluding humans, or one excluding all primates (Table 1). The results in Fig. 5a show how the accuracy of AlignFS and Genewise decreases as the databases diverge, with AlignFS more accurate at shorter distances. Notably Genewise produces accurate ORFs 60 % of the time, irrespective of the underlying database. Thus, AlignWise is not as affected by evolutionary distance as AlignFS, and out-performs both AlignFS and Genewise using more diverged databases. To assess how ESTscan responds to evolutionary distance we used the publically available HMMs from human, mouse and zebrafish. Although the number of models available is a limitation, only 25 % of ORFs processed by ESTscan perfectly align to the original coding sequence. As databases diverge, AlignFS and Genewise ORFs decrease in length, with AlignFS consistently finding shorter sequences (Fig. 5b). This highlights the AlignFS algorithm, designed to find accurate sequences at the expense of length. In contrast ORFs identified by ESTscan are consistently longer than the actual protein-coding regions.

**Fig. 6:**
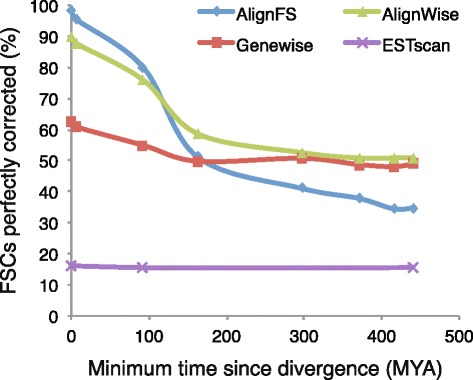
The accuracy of frame-shift corrections drops using divergent databases. For those ORFs with a frame-shift correction, the proportion that can be perfectly aligned to the original CDS are plotted. The accuracy of AlignFS drops as the minimum distance between the database and target species increases. By combining these results with Genewise, the drop is not as severe and remains above Genewise used individually

Many identified ORFs will not contain a frame-shift correction (FSC) and instead be truncated. We therefore analysed the quality of ORFs containing a FSC, identifiable from the log files. Using the whole RefSeq database, AlignFS accurately fixes 98 % of 784 frame-shift corrections (Fig. 6). Excluding human sequences from this database AlignFS perfectly corrects 95 % of the 765 FSCs. When using a BLAST database excluding all eutherian sequences this drops to 51 % (of 460 FSC). Genewise made 927 FSCs using the whole RefSeq database, of which only 580 (62 %) were accurate. However, this value is unaffected as the reference sequences diverge demonstrating Genewise is insensitive to evolutionary distance. By combining AlignFS and Genewise in AlignWise, we maximize the benefits of each of these approaches. Using a database that excludes all Eutherian sequences, AlignWise makes 507 FSCs of which 58 % are accurate. ESTscan, using a human HMM, attempted 644 FSCs, of which only 16 % were correct. Thus at close evolutionary distance AlignFS is the most accurate method of correcting frame-shifts. As evolutionary distance increases, combining these results with Genewise produces the best outcome.

**Fig. 7:**
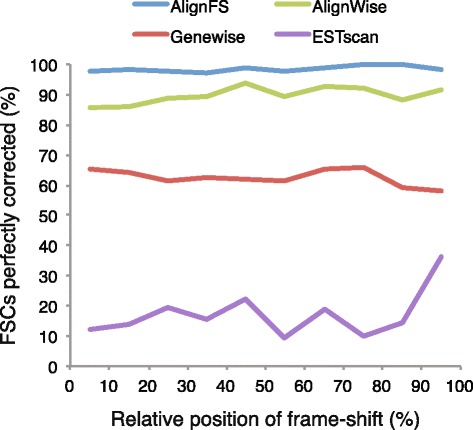
The accuracy of the ORFs is independent of frame-shift location. For those sequences with a frame-shift correction, the proportion of accurate ORFs is shown according to the relative location of the frame-shift within the CDS. AlignFS, Genewise and AlignWise were run using the RefSeq database, and ESTscan used the human HMM

We next assessed whether AlignWise is affected by the relative position of the frame-shift within the ORF (Fig. 7). Using the whole RefSeq database, AlignFS, Genewise and AlignWise show approximately the same proportion of accurate ORFs independent of the frame-shift location. ESTscan, using the human HMM, shows a small increase in accuracy when the frame-shift is located at the end of the coding-region.

### Running AlignWise on real data

To compare AlignWise and ESTscan on real data from a non-reference species we ran both programs on 1000 *Acipenser sinensis* ESTs; ESTscan found 498 ORFs using a zebrafish HMM, AlignWise identified 458 using the default BLAST database and 511 using the larger RefSeq database. ESTscan altered the sequence of 68 ESTs, while AlignWise corrected frame-shifts in 81 ESTs using the default database and 94 using the RefSeq database. Individually, AlignFS made 79 FSC using the default database and 84 using the RefSeq database, while Genewise made 94 and 111 FSC respectively. To compare the resulting ORFs we selected those sequences where at least one program made a frame-shift correction. We compared these ORFs against their putative Zebrafish homolog by a distance matrix using the GY94 codon substitution model in HyPhy [21, 22]. We excluded those where ORFs were identical, as well as those with a minimum distance greater than 2, as in [3]. Using the default database, AlignWise, AlignFS and Genewise behave similarly with 28, 27 and 29 of the ORFs having the shortest distance to Zebrafish respectively. Comparing the distance matrices produced using the RefSeq database, AlignWise outperforms the other programs as 42 of its ORFs have the shortest distance to zebrafish. This compares to 26 ORFs produced by Genewise, 17 AlignFS ORFs and 12 ORFs produced by ESTscan. Thus AlignWise out-performs AlignFS and Genewise individually as well as ESTscan using real EST data.

**Fig. 8:**
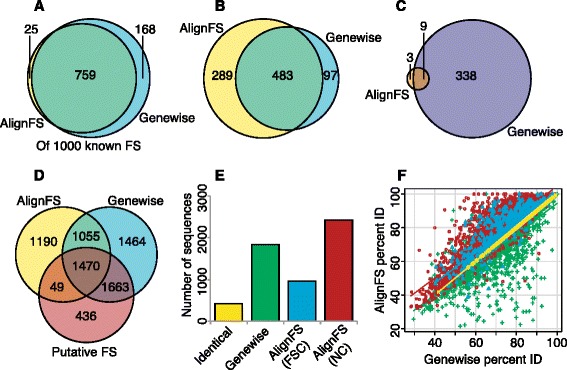
Comparing FSCs made by AlignFS, Genewise and AlignWise in human cDNAs and newt transcripts. **a**, **b** and **c**) Show results from the human cDNAs, using the RefSeq database, as shown in Table 3 and Fig. 6. **d**, **e** and **f** Show results from the newt transcripts. **a** Venn diagram of the number of FSCs made by AlignFS and Genewise. **b** The number of perfect FSCs made by AlignFS and Genewise. **c** Venn diagram of the number of incorrect FSCs made by AlignFS and Genewise. **d** Venn diagram of the number of FSCs made by AlignFS and Genewise intersecting with the putative frame-shifts identified previously [7]. **e** Illustrates the decisions made by AlignWIse for each of the 5,652 sequences that Genewise made FSCs in. Identical (yellow): Genewise and AlignFS make identical FSCs, Genewise (green): The Genewise FSC is selected, AlignFS(FSC)(blue): The AlignFS FSC is selected, AlignFS(NC)(red): The uncorrected AlignFS sequence is used.**f** For the proteins in (**e**), the percent identity against the original top BLASTx hit is compared for AlignFS and Genewise. Colours as in (**e**)

Finally we tested the ability of AlignWise to identify and correct frame-shifts within a de-novo assembled newt transcriptome generated by Looso and colleagues [7]. Using mass-spec proteomics the authors suggested 3,618 transcripts contain a putative frame-shift and confirmed this for a single sequence. We therefore asked how many of the original 14,471 transcripts were identified as protein-coding and containing frame-shifts using either AlignFS, Genewise or AlignWise. Using the RefSeq database, AlignFS identified 13,933 ORFs, of which 3,764 featured a FSC (Table 4). Genewise identified the same number of ORFs but made more FSCs, while AlignWise selected fewer frame-shift corrected sequences than either program made independently. For the 3,618 sequences previously identified as containing a putative frame-shift [7], 78 had no BLASTx result and so no ORF in either AlignFS, Genewise or AlignWise. AlignFS made a FSC in 1,519 of these sequences (Table 4), a further 1,823 used the top BLASTx hit as the alignment quality was not high enough to make a frame-shift correction. Genewise, which made more FSCs, altered 3,133 of the 3,618 transcripts. In the absence of a known reference it is impossible to determine which of these FSCs are accurate. However Genewise made more FSCs than AlignFS in our analysis of human cDNAs (Fig. 8a). In this analysis with a known reference we show the proportion of perfectly corrected FSCs is higher for AlignFS than Genewise (Fig. 8b) with Genewise making erroneous FSCs (Fig. 8c). It follows that the large proportion of Genewise FSCs made in the newt transcriptome, for which AlignFS is not making a FSC (Fig. 8d), may be incorrect. Indeed, for those sequences where Genewise makes a FSC, AlignWise typically selects the AlignFS protein without a FSC (Fig. 8e). Thus the final sequences selected by AlignWise maximize the percent identity to the reference protein demonstrating the ability of AlignWise to exploit the best performance of Genewise and AlignFS (Fig. 8f).

**Table 4:** Analysing the newt transcriptome

	Total ORFs			ORFs with putative frame-shift		
Program	Top HSP used	No changes made	FSC made	Top HSP used	No changes made	FSC made
AlignFS	5375	4793	3764	1823	198	1519
Genewise		8281	5652		407	3133
AlignWise	4216	6077	3639	1438	281	1821
ESTscan		8579	5589		694	2917

Furthermore, 891 of the newt frame-shifts identified by mass-spec are thought to contain multiple frame-shifts [7]. Of these, AlignWise selects 522 of the AlignFS sequences, 110 of which contain multiple FSCs. AlignWise selects 318 Genewise sequences, 284 of which contain multiple FSCs. A further 51 sequences were identical in both AlignFS and Genewise, 28 of which featured multiple corrections. Thus, AlignFS and Genewise are individually capable of identifying multiple frame-shifts within a single transcript. However, AlignWise continues to select a larger proportion of AlignFS than Genewise proteins. Along with our previous analyses, this demonstrates that AlignWise is capable of identifying and correcting frame-shifts within real de-novo assembled transcriptomes.

## Conclusions

AlignFS is a conservative frame-shift correction algorithm designed for large-scale phylogenetic analysis where high accuracy is preferable over a full length ORF. By combining this algorithm with Genewise, AlignWise is capable of producing accurate ORFs with low false positives across a wide range of evolutionary distances (summarized in Table 5). Increasing compute power and providing larger databases further improves the run time and accuracy of AlignWise. In our tests, AlignWise out-performs ESTscan at identifying biologically relevant protein-coding sequences and accurately correcting frame-shifts. Furthermore the AlignFS algorithm is accurate at short distances, and is able to improve Genewise ORFs using more distant homology. AlignWise thus exploits the best performance of AlignFS and Genewise to generate ORF nucleotide sequences corrected for potential frame-shifts from assembled transcripts, irrespective of their origin, and ESTs.

**Table 5:** A summary of ORF finding programs

Program	Finds ORFs	Outputs DNA sequences	Able to correct frame-shifts	Accurate at short evolutionary distances	Accurate at long evolutionary distances
AlignWise	Y	Y	Y	Y	Y
AlignFS	Y	Y	Y	Y	N
Genewise	Y	Y	Y	N	Y
ESTscan	Y	Y	Y	N	N
TransDecoder	Y	Y	N	N/A	N/A
OrfPredictor	Y	Y	N	N/A	N/A
MACSE	N^a^	Y	Y	N/A	N/A
BLASTx	Y	N	N	N/A	N/A
ESTwise	Y	N	Y	N/A	N/A

^a^We have indicated that MACSE was unable to find ORFs as it was unable to complete any analysis within a reasonable time frame

## Availability and requirements

**Project name:** AlignWise

**Project home page:**www.github.com/Looselab/AlignWise

**Operating systems:** UNIX and Linux

**Programming language:** Perl

**Other requirements:** BioPerl, BLAST+, MUSCLE

**License:** FreeBSD

**Any restrictions to use by non-academics:** none

## Abbreviations

ORF: Open reading frame
FSC: Frame-shift correction
